# Carbonized Nickel Complex of Sodium Pectate as Catalyst for Proton-Exchange Membrane Fuel Cells

**DOI:** 10.3390/membranes13070635

**Published:** 2023-06-30

**Authors:** Kirill V. Kholin, Aigul F. Sabirova, Danis M. Kadirov, Ayrat R. Khamatgalimov, Mikhail N. Khrizanforov, Irek R. Nizameev, Mikhail V. Morozov, Radis R. Gainullin, Timur P. Sultanov, Salima T. Minzanova, Eugene S. Nefed’ev, Marsil K. Kadirov

**Affiliations:** 1 Arbuzov Institute of Organic and Physical Chemistry, FRC Kazan Scientific Center, Russian Academy of Sciences, Kazan 420088, Russia; kholin06@mail.ru (K.V.K.); aigul84saf@mail.ru (A.F.S.); ayrat_kh@iopc.ru (A.R.K.); khrizanforov@gmail.com (M.N.K.); inizameyev@iopc.ru (I.R.N.); radis.g@mail.ru (R.R.G.); sultanovtp05@mail.ru (T.P.S.); minzanova@iopc.ru (S.T.M.); 2 Department of Physics, Kazan National Research Technological University, Kazan 420015, Russia; daniskadirov@gmail.com (D.M.K.); nefedev1947@gmail.com (E.S.N.); 3 A.M. Butlerov Chemistry Institute, Kazan Federal University, Kremlevskaya Str. 18, Kazan 420008, Russia; 4 Department of Nanotechnology in Electronics, Kazan National Research Technical University named after A.N. Tupolev—KAI, Kazan 420111, Russia; misha617@mail.ru

**Keywords:** carbonization, coordination biopolymers, proton-exchange membrane fuel cell, oxygen-reduction reaction, hydrogen-oxidation reaction, nickel complex, sodium pectate, 2/4-electron transfer

## Abstract

Sodium pectate derivatives with 25% replacement of sodium ions with nickel ions were obtained by carbonization to temperatures of 280, 550, and 800 °C, under special protocols in an inert atmosphere by carbonization to temperatures of 280, 550, and 800 °C. The 25% substitution is the upper limit of substitution of sodium for nickel ions, above which the complexes are no longer soluble in water. It was established that the sample carburized to 550 °C is the most effective active element in the hydrogen-oxidation reaction, while the sample carbonized up to 800 °C was the most effective in the oxygen-reduction reaction. The poor performance of the catalytic system involving the pectin coordination biopolymer carbonized up to 280 °C was due to loss of proton conductivity caused by water removal and mainly by two-electron transfer in one catalytic cycle of the oxygen-reduction reaction. The improved performance of the system with coordination biopolymer carbonized up to 550 °C was due to the better access of gases to the catalytic sites and four-electron transfer in one catalytic cycle. The (Ni-NaPG)_800C_ sample contains metallic nickel nanoparticles and loose carbon, which enhances the electrical conductivity and gas capacity of the catalytic system. In addition, almost four-electron transfer is observed in one catalytic cycle of the oxygen-reduction reaction.

## 1. Introduction

The use of fossil energy has led to significant damage to the natural environment and human health due to greenhouse gases, toxic smoke, and dust. Therefore, there is an urgent need to replace traditional fuel vehicles with electric vehicles that can be charged using renewable energy sources [1]. Renewable energy has become the main energy source nowadays. However, its intermittence and instability pose significant challenges to the stable operation of the power system, creating temporal and spatial gaps between the availability of energy and its consumption by end-users [2]. Energy storage and conversion technology, such as PEMFCs, are crucial for achieving stable and efficient renewable energy. However, the high cost of precious-metal platinum catalysts is the biggest problem and challenge facing PEMFCs, as it limits their large-scale application [3].

The low/non-platinum catalyst proposed in this paper has important scientific significance due to the high cost of platinum catalysts in PEMFCs. Additionally, biomaterials are biodegradable, biocompatible, and environmentally friendly [4]. Natural biomaterials can be extracted without the need for complex chemical synthesis methods. Promising biomaterials have been used in electronic and optoelectronic devices, some of which have potential consumer value for non-volatile transistor memory and write-once–read-multiple memory applications [5,6,7].

Research has shown that sodium pectate with a 25% substitution of sodium ions for nickel ones can be used as ORR [8] and HOR [9] catalysts in PEMFCs as an active material in non-platinum catalytic systems. Recently, varying the concentration of Ni(II) ions in nickel complexes of sodium pectate [10] allowed researchers to find the optimal concentration of the transition metal to improve the characteristics of the studied PEMFCs.

Porous carbon is important in various applications, such as fuel-cell electrocatalysts, electrochemical decomposition of water, supercapacitors, lithium-ion batteries, hydrogen storages, etc., due to its friability and large specific surface area, high electrical conductivity, and stability of electrochemical characteristics in both acidic and alkaline media [11,12,13,14,15]. The development of advanced nanostructured porous carbon materials with customizable micro/nanostructure, porosity, crystallinity, and controlled composition has been a focus of research in recent decades [16,17,18,19,20]. Porous carbon materials can be obtained by carbonization/activation, using renewable biomass precursors such as sugars, polysaccharides, and lignocelluloses as a carbon source [21,22]. These natural biopolymers offer advantages, such as being green and sustainable carbon materials for energy applications [23,24,25,26,27,28,29,30,31,32,33,34].

This article shows that carbonized complexes of natural pectin polysaccharides are suitable candidates for the role of promising non-platinum catalytic materials in the energy sector.

## 2. Results and Discussion

### 2.1. Synthesis of Samples by Carbonization

**Figure 1:**
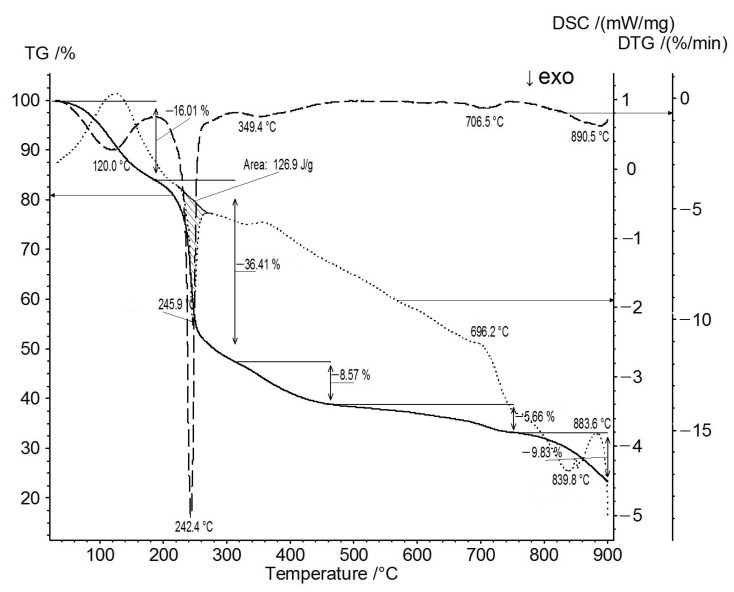
TG/DSC curves of Ni(25%)-NaPG in an argon atmosphere: TG—solid line; DTG—dotted line; and DSC—dashed line.

The samples were synthesized by the carbonization of sodium polygalacturonate, 25% of which was replaced by nickel [Ni(25%)-NaPG], in an inert atmosphere (Ar), to various temperatures. The TG/DSC curve of Ni(25%)-NaPG (shown in Figure 1) indicates that the thermogram has five noticeable weight-loss stages, namely at 120.0 °C, 242.4 °C, 349.4 °C, 706.5 °C, and 890.5 °C. The first stage, at 120 °C, with the corresponding endothermic peak, is estimated at ~16% weight loss. The second stage of weight loss on the TG curve (~36%) is observed at 242.4 °C and is accompanied by an exothermic peak at 246 °C, with an enthalpy estimated at ~126.9 J/g. The fifth stage of weight loss (~9.8%), at 890.5 °C, is accompanied by an exothermic peak. The maximum absorption peaks at 120.0 °C and 242.4 °C are associated with significant variations in the chemical structure, namely the loss of water in the first stage and the decarboxylation and decomposition in the second stage.

After the second, third, and fourth mass losses, temperatures of 280 °C, 550 °C, and 800 °C were chosen, up to which carbonization was carried out according to special protocols, the programs of which are listed in Table 1. The sample prepared in an argon atmosphere according to Program (1) is denoted as (Ni-NaPG)_280C_, the one prepared according to Program (2) is (Ni-NaPG)_550C_, and that prepared according to Program (3) is (Ni-NaPG)_800C_.

### 2.2. Transmission Electron Microscopy

**Figure 2:**
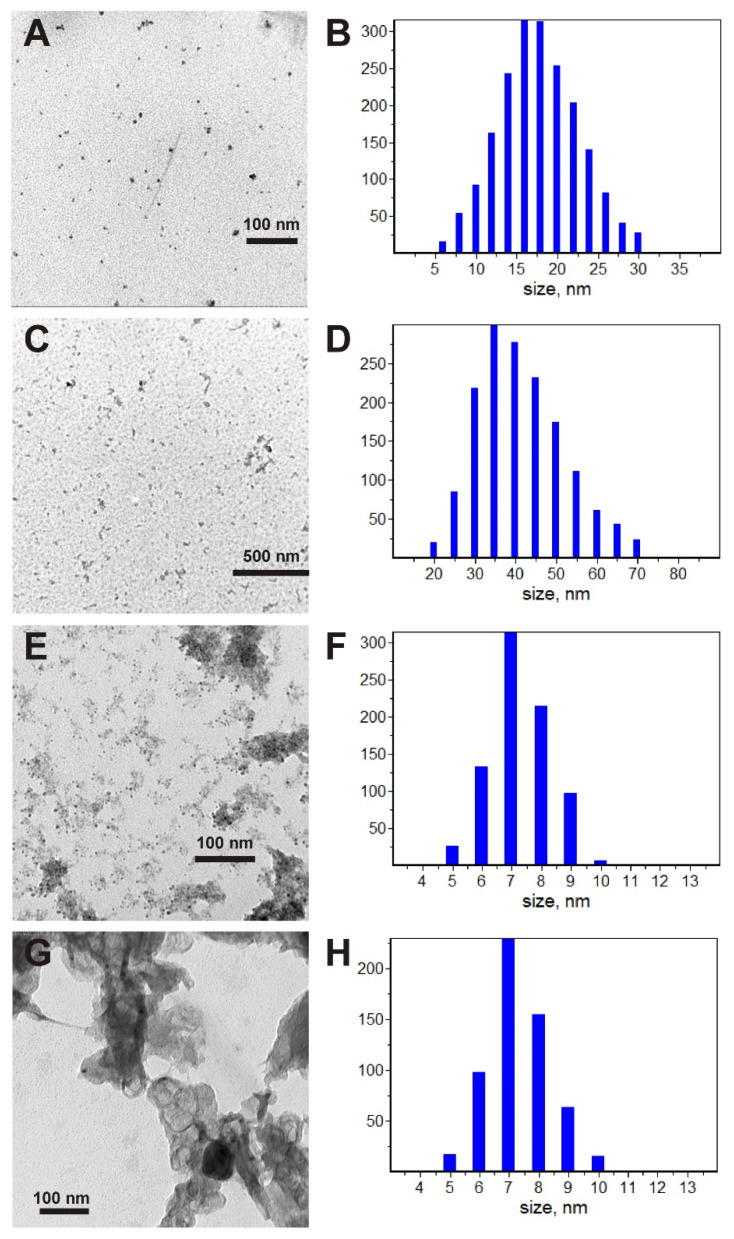
TEM images (left column) and horizontal particle size distributions (right column) for Ni(25%)-NaPG (**A**,**B**), (Ni-NaPG)_280C_ (**C**,**D**), (Ni-NaPG)_550C_ (**E**,**F**), and (Ni-NaPG)_800C_ (**G**,**H**).

Figure 2 shows the TEM images and distributions of particles by horizontal sizes. The Ni(25%)-NaPG sample contains long aggregates with an aspect ratio of more than 10:1, as seen in the structure of the TEM image (Figure 2A). A detailed study of long aggregates showed the presence of densely packed small particles with sizes of 3–5 nm in a matrix of a different compositions (presumably a metal in an organic cellular matrix). The size distribution of aggregates (Figure 2B) does not take into account the internal structure of aggregates, and small particles are not included in the calculation. The average particle size in the horizontal plane is 17 nm.

Based on the TEM images shown in Figure 2C, we can see that the sample carbonized to 280 °C contains aggregates similar to those of Ni(25%)-NaPG. However, small metal particles are now densely interspersed both in long aggregates and in metal–organic systems. The size distribution (Figure 2D) shows a twofold increase in the average size (35 nm) of aggregates.

The TEM images of the (Ni-NaPG)_550C_ sample, as shown in Figure 2E, consist of many individual particles, with sizes ranging from 5 to 10 nm. These particles occur separately in some regions and in combination with an environment of a less dense substance (from the point of view of electron beam penetration) in others. This environment behaves differently in different areas: in some areas, it does not have a clear shape and resembles the structures observed for pectin; meanwhile, in others, it takes the form of long and highly branched systems with many channels that are filled with individual small particles mentioned earlier. The average particle size is approximately 7 nm (Figure 2F).

According to Figure 2G of the TEM images, the (Ni-NaPG)_800C_ sample is similar in many aspects to the previous one. It contains a large number of separate spherical particles with sizes ranging from 5 to 10 nm, and long filamentous structures are also visible. However, there is a new type of substance that tightly sticks around the filamentous structures and is located in large clots in other areas. This is likely due to the carbonization of the organic component, which results in the formation of carbon. The particle size distribution over the carbon-free areas (Figure 2H) strongly resembles the distribution for the (Ni-NaPG)_550C_ sample.

Thus, based on the information provided, we can conclude that nickel nanoparticles with an average size of 7 nm are formed in samples carbonized at 550 and 800 °C. In the second case, the nickel nanoparticles are formed with carbon. However, in the sample (Ni-NaPG)_800C_, the nickel nanoparticles have a distinct metallic crystalline phase.

### 2.3. Specific Surface Area

Nitrogen adsorption and desorption isotherms were measured at liquid nitrogen temperature, using an Autosorb iQ MP analyzer (Quantachrome), as shown in Table 2. Before the measurement, the samples were degassed at a temperature of 100 °C, at a pressure of about 1 Pa, and for a degassing time of 13 h. The specific surface of the studied samples increases with an increase in carbonization temperatures, and for (Ni-NaPG)_800C_, it increases by more than two orders of magnitude compared to other samples and reaches a value of 132 m^2^/g.

The reason for the increase in the specific surface area of the studied samples with an increase in carbonization temperatures is due to the formation of carbon at high carbonization temperatures, as previously mentioned in Section 2.2. Additionally, the resulting carbon material has a large specific surface area, which also contributes to this phenomenon. It is important to note that this statement is accurate.

### 2.4. Cyclic Voltammetry (CV) of Ni(20%)-NaPG Solid-Phase Samples

**Figure 3:**
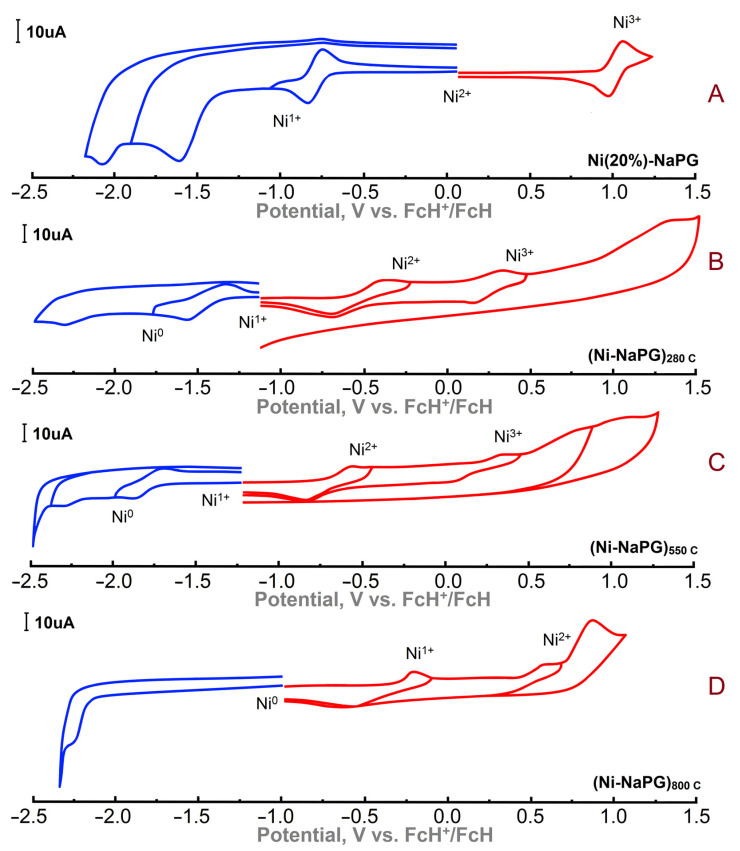
Cyclic voltammograms of the coordination biopolymer Ni(25%)-NaPG (**A**) and its carbonized derivatives (Ni-NaPG)_280C_ (**B**), (Ni-NaPG)_550C_ (**C**), and (Ni-NaPG)_800C_ (**D**). CPE, CH_3_CN, and 10^−1^ M Bu_4_NBF_4_; reference electrode—Fc/Fc^+^.

The electrochemical properties of the nickel–polymer complex Ni(25%)-NaPG and samples of this coordination biopolymer preliminarily carbonized according to special protocols up to temperatures of 280, 550, and 800 °C were studied by solid-phase electrochemistry (Figure 3 and Table 3). Thus, in the Ni(25%)-NaPG complex, the nodal nickel ions have a charge of 2^+^. During electrochemical reduction, a reversible transition to Ni(I) is observed, and during oxidation, a reversible transition to Ni(III) is observed too (Figure 3A).

According to the electrochemical properties of the (Ni-NaPG)_280 C_ sample, the initial state of the metal site is Ni(I) ion, as shown in Figure 3B. The first wave during reduction is characterized by a reversible peak and corresponds to the transition of Ni(I) to Ni(0). Further irreversible reduction is characterized by the formation of the radical anion of the ligand itself, which is clearly seen in the CV curve. Upon oxidation, the complexes exhibit reversible stepwise transitions of Ni(I) to Ni(II) and Ni(II) to Ni(III). Oxidation of the ligand in the study area is not observed because, after the carbonization of the initial sample, the hydroxy groups of the complex are no longer present.

The redox properties of a sample carbonized to a temperature of 550 °C were studied under similar conditions (Figure 3C). The initial state of the metal site also refers to the Ni(I) ion. The reduction potential corresponding to the Ni(I) to Ni(0) transition shifts by 0.33 V to a more negative region. The observed effect can be characterized by a structural change in the polymer matrix. The oxidation of the complex is also accompanied by reversible stepwise transitions of Ni(I) to Ni(II) and Ni(II) to Ni(III).

In the (Ni-NaPG)_800C_ sample (Figure 3D), the initial state of the coordination site is Ni(0), which indicates that the resulting nanoparticles are of a metallic nature. Sample oxidation is characterized by reversible stepwise transitions of Ni(0) to Ni(I) and Ni(I) to Ni(II).

### 2.5. ORR Kinetics Study on a Rotating Disk Electrode Using the Koutecký–Levich Method

The kinetics of the oxygen electroreduction reaction on a rotating disk electrode were studied using the Koutecký–Levich equation [35,36]:

1/I = 1/i_K_ + 1/i_D_ = 1/i_K_ + 1/Bnω^0.5^, (1)

where B = 0.62FD^2/^ υ^−1/6^c; i is the current on the disk electrode; i_K_ is the kinetic current; i_D_ is diffusion current; ω is the speed of rotation of the disk electrode (rad/s); n is the number of electrons involved in the electrochemical reaction; F is the Faraday constant, C/mol; D is the diffusion coefficient, cm^2^/s; υ is the kinematic viscosity of the electrolyte, cm^2^/s; and c is the concentration of oxygen in the solution. For our system, the parameters given in Table 4 were used, and F = 96,484.6 C/mol.

The recording speed is 50 mV/s, the working electrode is glassy carbon (S = 0.0707 cm^2^), and the electrolyte is 0.5 M H_2_SO_4_.

**Figure 4:**
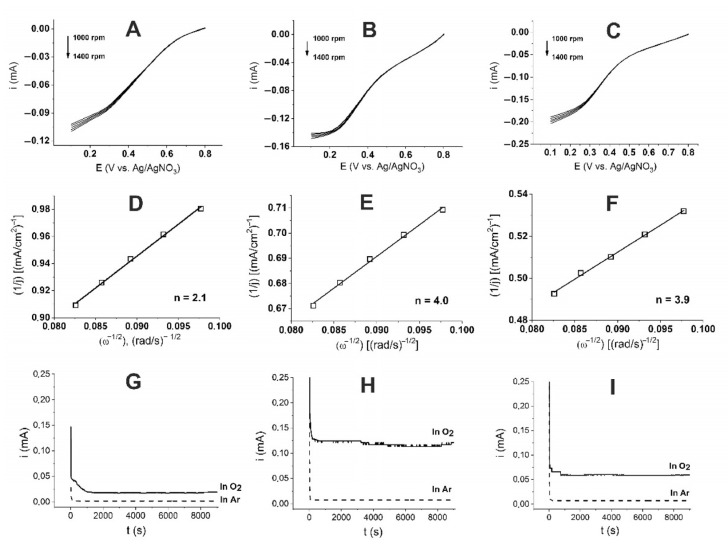
The line CVs (**A**–**C**) measured at different speeds of the RDE, the dependences of Koutecký–Levich at 0.3 V (**C**–**F**), corresponding to the (Ni-NaPG)_280C_ (**A**,**D**), (Ni-NaPG)_550C_ (**B**,**E**), and (Ni-NaPG)_800C_ (**C**,**F**) samples in 0.5 M H_2_SO_4_ solution. Potentiostatic stability tests of the (Ni-NaPG)_280C_/C (**G**), (Ni-NaPG)_550C_/C (**H**), and (Ni-NaPG)_800C_/C (**I**) catalyst on a CC electrode (S = 0.0707 cm^2^) in 0.5 M H_2_SO_4_ for 9000 s in solutions saturated with oxygen (solid thick curve) and argon (dashed curve) at the same applied potential (E = 0.3 V vs. Ag/AgNO_3_), with 0.05 mg of the catalyst.

Linear voltammograms of O_2_ reduction, using a rotating disk glassy carbon electrode modified with the studied catalysts in 0.5 M aqueous oxygen-saturated H_2_SO_4_ solution (Figure 4A–C), were used to study the ORR mechanism. The sloped Koutecký–Levich straight lines (Figure 4D–F) show that, for the (Ni-NaPG)_280C_ catalyst, the number of electrons transferred in one ORR catalytic cycle is 2.1; for the (Ni-NaPG)_550C_, it is 4; and for (Ni-NaPG)_800C_, it is 3.9.

### 2.6. Electrochemical Stability during ORR in Acidic Solution

Figure 4G–I show the results of the tests for the potentiostatic stability of catalysts on a CC electrode (S = 0.0707 cm^2^) in 0.5 M H_2_SO_4_ for 9000 s in solutions saturated with oxygen (solid curve) and argon (dashed curve) at the same applied potential E = 0.3 V with 0.05 mg of catalyst. The current in the argon atmosphere stabilized at a minimum value of 1 μA, while its value in an oxygen atmosphere was 13.5 μA for (Ni-NaPG)_280C_ (Figure 4G). For (Ni-NaPG)_550C_ (Figure 4H), these values are 7 and 120 μA, respectively. For (Ni-NaPG)_800C_ (Figure 4I), the values are 2 and 60 µA, respectively.

This implies that the studied catalysts effectively stabilize the oxygen reduction on the electrode surface, with the (Ni-NaPG)_550C_ catalyst exhibiting twice the activity of (Ni-NaPG)_800C_ and approximately nine times more activity than (Ni-NaPG)_280C_.

### 2.7. PEMFC Tests

**Figure 5:**
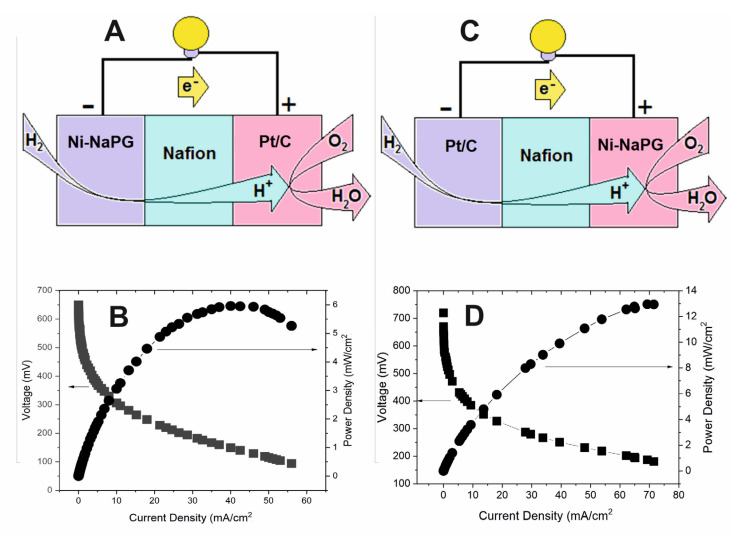
Representations (**A**,**C**) of the H_2_/O_2_ PEMFC and polarization and power curves at 80 °C for MEAs (Ni-NaPG)_550C_/Nf/Pt (**B**) and Pt/Nf/(Ni-NaPG)_800C_ (**D**).

Membrane-electrode assemblies (MEAs) were designed and tested using the studied carbonized-nickel-substituted biopolymers as cathode or anode electrocatalyst and commercial Pt/C as anode or cathode electrocatalyst, respectively, with a loading of 1 mg/cm^2^ (Table 5). Of the MEAs with newly synthesized carbonized-nickel pectates on the anode side, (Ni-NaPG)_550C_/Nf/Pt demonstrates (Table 5, Entry 9; Figure 5B) the highest performance for the hydrogen oxidation reaction (HOR), with a maximum current density of 56 mA/cm^2^ and power of 5.96 mW/cm^2^, while the open-circuit voltage (OCV) is the smallest of the three anode catalysts studied—650 V vs. 723 V for (Ni-NaPG)_280C_/Nf/Pt, and 847 V for (Ni-NaPG)_800C_/Nf/Pt. Of the newly developed catalytic systems for the ORR, Nf/Pt/(Ni-NaPG)_800C_ demonstrates the maximum diagnostic characteristics: HXX—670 mV; maximum specific current density—71.5 mA/cm^2^; and power—13 mW/cm^2^.

In the series of ORR catalysts based on nickel coordination biopolymers, the complex carbonized up to 280 °C worsens the diagnostic characteristics of PEMFC: OCV—from 710 mV to 530 mV; maximum specific current density—from 59 to 10.86 mA/cm^2^; and power—from 5.9 to 1.67 mW/cm^2^. One of the possible explanations is a decrease in the proton conductivity [37,38] of the coordination compound due to the loss of water due to heating above 100 °C during carbonization. The improvement in the performance of PEMFC upon going to the MEA Pt/Nf/(Ni-NaPG)_550C_ can be explained by the escape of hydrogens at these carbonization temperatures and the appearance of a better access of gases to the Ni^+^ catalytic sites. In (Ni-NaPG)_800C_, metallic nickel nanoparticles and loose carbon are around the system. The catalytic activity of ORR also depends on the number of transferred electrons in one catalytic cycle. As mentioned above, for (Ni-NaPG)_280C_, the number of transferred electrons in one catalytic cycle of ORR is 2.1; for (Ni-NaPG)_800C_, it is 3.9; and for (Ni-NaPG)_550C_, it is 4. The fact that the specific diagnostic characteristics of PEMFC based on the last two compounds are 6–7 times higher than those of the first one is quite expected from purely electrochemical kinetic studies.

Attention is drawn to the fact that, through the carbonization of the Ni(25%)-NaPG sample, it was possible to achieve characteristic values in the order of magnitude of the leader from a series [10,24] of 5, 10, 15, 20, and 25% substitution of sodium with nickel—Ni(20%)-NaPG (items 1 and 6). The strategy developed in this work on the carbonization of Ni(25%)-NaPG allows us to hope for a further improvement in the characteristics of nonplatinum catalytic systems through the carbonization of coordination biopolymers—leaders.

## 3. Methods

### 3.1. TG/DSC–FTIR

The thermal decomposition was studied by simultaneous thermal analysis (thermogravimetry/differential scanning calorimetry combined with FTIR spectroscopy TG/DSC–FTIR), and the variation of the sample mass as a function of temperature and the corresponding heats were recorded. A coupled system comprising a NETZSCH STA449-F3 TG/DSC instrument and a Bruker Tensor 27 FTIR spectrometer was used. The sample (~54 mg) was placed in an Al_2_O_3_ crucible with a perforated lid and heated from room temperature to 900 °C, together with an empty crucible as the reference. The TG/DSC measurement was carried out at a heating rate of 10 K/min in an argon flow of 50 mL/min. The resolution of the Tensor 27 spectrophotometer was 4 cm^−1^. The gas cell and the transport line between TG/DSC and the FTIR spectrometer were heated to 200 and 195 °C, respectively.

### 3.2. TEM

The transmission electron microscopy images were obtained with a Hitachi HT7700, Japan. The images were acquired at an accelerating voltage of 100 kV. Samples were ultrasonicated in water for 10 min and then dispersed onto a 300-mesh copper grid with continuous carbon–formvar support film.

### 3.3. Specific Surface Area

The specific surface area was measured on an Autosorb iQ MP (Quantachrome) device for the adsorption of gaseous nitrogen, using the Brunauer, Emmett, and Teller method (BET method).

### 3.4. Electrochemistry

Electrochemical measurements were performed on a BASiEpsilonE2P electrochemical analyzer (USA). The program handles wave Epsilon-EC-USB-V200. A conventional three-electrode system was used with glassy carbon for solutions or a carbon paste electrode (CPE) for powder samples as the working electrode, the Ag/AgCl (0.01 M) electrode as the reference electrode, and a Pt wire as the counter electrode. Then, 0.1 M Et_4_NBF_4_ was used as the supporting electrolyte for the determination of current−voltage characteristics. Acetonitrile was distilled over P_2_O_5_ and KMnO4 and then over molecular sieves. After purification, the solvent was stored under dry argon. Used as a base salt, Et_4_NBF_4_ was recrystallized from ethanol and dried in a vacuum chamber at 100 °C for 2 days. To study powder samples, a modified CPE working electrode was used. Its preparation was as follows: the carbon particles/phosphonium salt (dodecyl(tri-*tert*-butyl)phosphonium tetrafluoroborate) composite electrode was prepared by grinding a mixture of graphite powder and phosphonium salt in a ratio of 90/10 (*w*/*w*) in a mortar to give the homogeneous mass. A modified electrode was made in a similar manner, except that a part (ca. 5%) of graphite powder was replaced by samples under investigation. A portion of the resulting paste was packed firmly into the cavity (3 mm in diameter) of a Teflon holder.

### 3.5. Fuel Cell Tests

Polarization curves were obtained using the mechanical test station ElectroChem (United States) with the gas flow and pressure control system MTS-A-150 and the electronic load unit ECL-150. The MEA was tested in a standard 1 cm^2^ PEMFC of ElectroChem Ink (US, catalogue number—FC-01-02), as shown in Supplementary Figure S3. During the measurement of the polarization curves, the load was gradually increased until the maximum voltage (open-circuit voltage) was reached. The cell temperature was 80 °C. The flow rates of hydrogen and oxygen were at 1.7 mL s^−1^ and 3.3 mL s^−1^, respectively. Anode and cathode gases were humidified at 25 °C. The backpressure of gases is 2.0 and 3.4 atm on the anode and cathode sides of the cell, respectively.

The catalytic ink was prepared by adding 4 mg of organometallic catalyst and 40 mg of Vulcan XC-72 to a mixture of 1.5 mL IPA and 1.5 mL deionized water. The resulting ink was sonicated for 15 min, followed by the addition of 320 µL of 10 wt% Nafion^®^ solution (Aldrich), and sonicated again for an hour. The E-TEK (Pt20/C) catalytic ink was prepared in a similar manner, where 20 mg of E-TEK was added to 8.4 mL IPA, sonicated for 15 min, and then mixed with 81 µL of 10 wt% Nafion^®^ solution via sonication. The inks were deposited on a Sigracet^®^ 25CC carbon paper gas-diffusion layer, and the MEA was obtained by hot-pressing GDLs on both sides of the Nafion^®^ 212 membrane at 90 °C, with a load of about 300 lbs, for 4 min. The surface concentration of the nickel catalyst was 1 mg/cm^2^, while that of Pt was 1 mg/cm^2^ (5 mg Pt20/C on 1 cm^2^). The area of the MEA was 1 cm^2^, and the humidity of the supplied gases was not regulated. These were the experimental conditions used in the study. The conclusions drawn from these experiments are not provided in this excerpt.

## 4. Conclusions

The studied samples were synthesized using the carbonization method, with special protocols of sodium polygalacturonate, where 25% of it was replaced by nickel [Ni(25%)-NaPG], in an inert atmosphere, at various temperatures. The study of the kinetics of the oxygen-reduction reaction on a rotating-disk electrode using the Koutecký–Levich method revealed that the number of electrons transferred per one ORR catalytic cycle was 2.1 for the (Ni-NaPG)_280C_ catalyst, 4 for (Ni-NaPG)_550C_, and 3.9 for (Ni-NaPG)_800C_. The newly developed MEA (Ni-NaPG)_550C_/Nf/Pt catalytic system exhibited the highest density diagnostic characteristics for the hydrogen oxidation reaction, with a maximum current density of 56 mA/cm^2^ and a power of 5.96 mW/cm^2^. On the other hand, the MEA Nf/Pt/(Ni-NaPG)_800C_ showed the best performance in the oxygen-reduction reaction, with an OCV of 670 mV, maximum specific current density of 71.5 mA/cm^2^, and power of 13 mW/cm^2^.

The developed strategy for processing coordination biopolymers makes it possible to improve the performance of the leading samples of the non-platinum catalysts.

## Figures and Tables

**Table 1:** Temperature programs for carbonization of samples in an argon atmosphere.

Program (1)—280 °C	Program (2)—550 °C	Program (3)—800 °C
t_room_–140 °C–10 °C/min Isotherm: 30 min 140–150 °C–2 °C/min Isotherm: 30 min 150–195 °C–1 °C/min Isotherm: 30 min 195–230 °C–1 °C/min Isotherm: 30 min 230–250 °C–1 °C/min Isotherm: 30 min 250–280 °C–1 °C/min Isotherm: 30 min	Program (1) + 280–300 °C–1 °C/min Isotherm: 30 min 300–350 °C–1 °C/min Isotherm: 30 min 350–550 °C–5 °C/min Isotherm: 30 min	Program (2) + 550–800 °C–5 °C/min Isotherm: 30 min

**Table 2:** Specific surface area of samples measured on the Autosorb iQ MP analyzer (Quantachrome) by nitrogen gas adsorption, using the Brunauer, Emmett, and Teller method (BET method).

Sample	Sample Degassing Conditions			Specific Surface According to BET, m^2^/g
	Time, Hour	Temperature, °C	Degree of Evacuation, Pa	
(Ni-NaPG)_280C_	13	100	1	0.45
(Ni-NaPG)_550C_	13	100	1	1.19
(Ni-NaPG)_800C_	13	100	1	132

**Table 3:** Redox peaks and potentials of (Ni-NaPG)_280C_, (Ni-NaPG)_550C_, and (Ni-NaPG)_800C_ vs. Fc/Fc^+^. CPE, CH_3_CN, and 10^−1^M Bu_4_NBF_4_.

Sample	Reduction		Oxidation		
	*^1^E_p_^c^/E_p_^a^_,_ V* *Semidif, V*	*^2^E_p_^c^/E_p_^a^_,_ V* *Semidif, V*	*^1^E_p_^a^/E_p_^c^_,_ V* *Semidif, V*	*^2^E_p_^a^/E_p_^c^_,_ V* *Semidif, V*	*^3^E_p_^a^/E_p_^c^_,_ V* *Semidif, V*
(Ni-NaPG)_280C_	−1.61/−1.35 −1.49	−2.32 (irrev) −2.19	−0.35/−0.70 −0.54	0.31/0.17 0.22	
(Ni-NaPG)_550C_	−1.78/−1.88 −1.82	−2.32 (irrev) −2.33	−0.38/−0.80 −0.61	0.35/0.01 0.17	0.75 (irrev) 0.72
(Ni-NaPG)_800C_	−2.22 (irrev) −2.20		−0.22/−0.65 −0.41	0.60/0.35 0.49	0.85 (irrev) 0.82

**Table 4:** Parameter values for the Koutecký–Levich equation.

Electrolyte	D, cm^2^/s	υ, cm^2^/s	CO_2_, mol/cm^3^	B, mAs^0.5^cm^−2^
0.5 M H_2_SO_4_	1.8 × 10^−5^	0.01	1.13 × 10^−6^	0.4

**Table 5:** Open-circuit voltage (OCV), current density, and maximum power density generated by various PEMFCs based on carbon black (Vulcan XC-72) and carbon-black-supported Pt.

Entry	Anode Catalyst	Cathode Catalyst	OCV (mV)	Maximum Current Density (mA cm^−2^)	Maximum Power Density (mW cm^−2^)
1 ^[b]^	Pt	Ni(20%)-NaPG/C	602	57.8	10.58
2 ^[c]^	Pt	Ni(25%)-NaPG/C	710	59	5.9
3	Pt	(Ni-NaPG)_280C_/C	530	10.86	1.67
4	Pt	(Ni-NaPG)_550C_/C	663	65.2	12.5
5	Pt	(Ni-NaPG)_800C_/C	670	71.5	13
6 ^[b]^	Ni(20%)-NaPG/C	Pt	929	23.21	6.03
7 ^[d]^	Ni(25%)-NaPG/C	Pt	960	5.2	1.5
8	(Ni-NaPG)_280C_/C	Pt	723	1.45	0.31
9	(Ni-NaPG)_550C_/C	Pt	650	56	5.96
10	(Ni-NaPG)_800C_/C	Pt	847	1.42	0.34
11	Pt	Pt	1	1280	324

^[b]^ From [10]. ^[c]^ From [8]. ^[d]^ From [9].

## Data Availability

Not applicable.

## Supplementary Materials

The following supporting information can be downloaded at https://www.mdpi.com/article/10.3390/membranes13070635/s1. Figure S1. IR spectra of the pectin, sodium polygalacturonate (NaPG) and sodium polygalacturonate complexes [Ni(20%)-NaPG], [Ni(25%)-NaPG] with 20% and 25% substitution of sodium with nickel, consequenly. Figure S2. The scheme of electrochemical set-up. 1—computer, 2—potentiostat Elins P-20x, 3—rotating disc electrode—Basi RDE-2, 4—vessel with inert gas (Ar), 5—vessel with oxygen, 6—valve of microadjusing, 7—counter electrode, 8—capillary for gas introducing, 9—working electrode, 10—reference electrode. Figure S3. Scheme and appearance of the standard PEMFC used in the tests.
