# Supplementary material for: Carbonized Nickel Complex of Sodium Pectate as Catalyst for Proton-Exchange Membrane Fuel Cells
Source: Membranes (Basel). 2023 Jun 30;13(7):635. doi: 10.3390/membranes13070635 (PMC10384383; doi:10.3390/membranes13070635)
Supplement: Supplementary file 1 [file membranes-13-00635-s001.zip › membranes-2383934-supplementary.pdf]

Supplementary Material

Carbonized Nickel Complex of Sodium Pectate as Catalyst for Proton-Exchange Membrane Fuel Cells

Kirill V. Kholin [1,2], Aigul F. Sabirova [1,2], Danis M. Kadirov [2], Ayrat R. Khamatgalimov [1], Mikhail N. Khrizanforov [1,3], Irek R. Nizameev [1,2,4], Mikhail V. Morozov [4], Radis R. Gainullin [1,2], Timur P. Sultanov [1,2], Salima T. Minzanova [1], Eugene S. Nefed'ev [2] and Marsil K. Kadirov [1,2,*]

[1] Arbuzov Institute of Organic and Physical Chemistry, FRC Kazan Scientific Center, Russian Academy of Sciences, Kazan 420088, Russia; kholin06@mail.ru (K.V.K.); aigul84saf@mail.ru (A.F.S.); ayrat_kh@iopc.ru (A.R.K.); khrizanforov@gmail.com (M.N.K.); inizameyev@iopc.ru (I.R.N.); radis.g@mail.ru (R.R.G.); sultanovtp05@mail.ru (T.P.S.); minzanova@iopc.ru (S.T.M.)

[2] Department of Physics, Kazan National Research Technological University, Kazan 420015, Russia; daniskadirov@gmail.com (D.M.K.); nefedev1947@gmail.com (E.S.N.)

[3] A.M. Butlerov Chemistry Institute, Kazan Federal University, Kremlevskaya Str. 18, Kazan 420008, Russia

[4] Department of Nanotechnology in Electronics, Kazan National Research Technical University named after A.N. Tupolev—KAI, Kazan 420111, Russia; misha617@mail.ru

* Correspondence: kamaka59@gmail.com; Tel.: +7-(927)-417-45-59

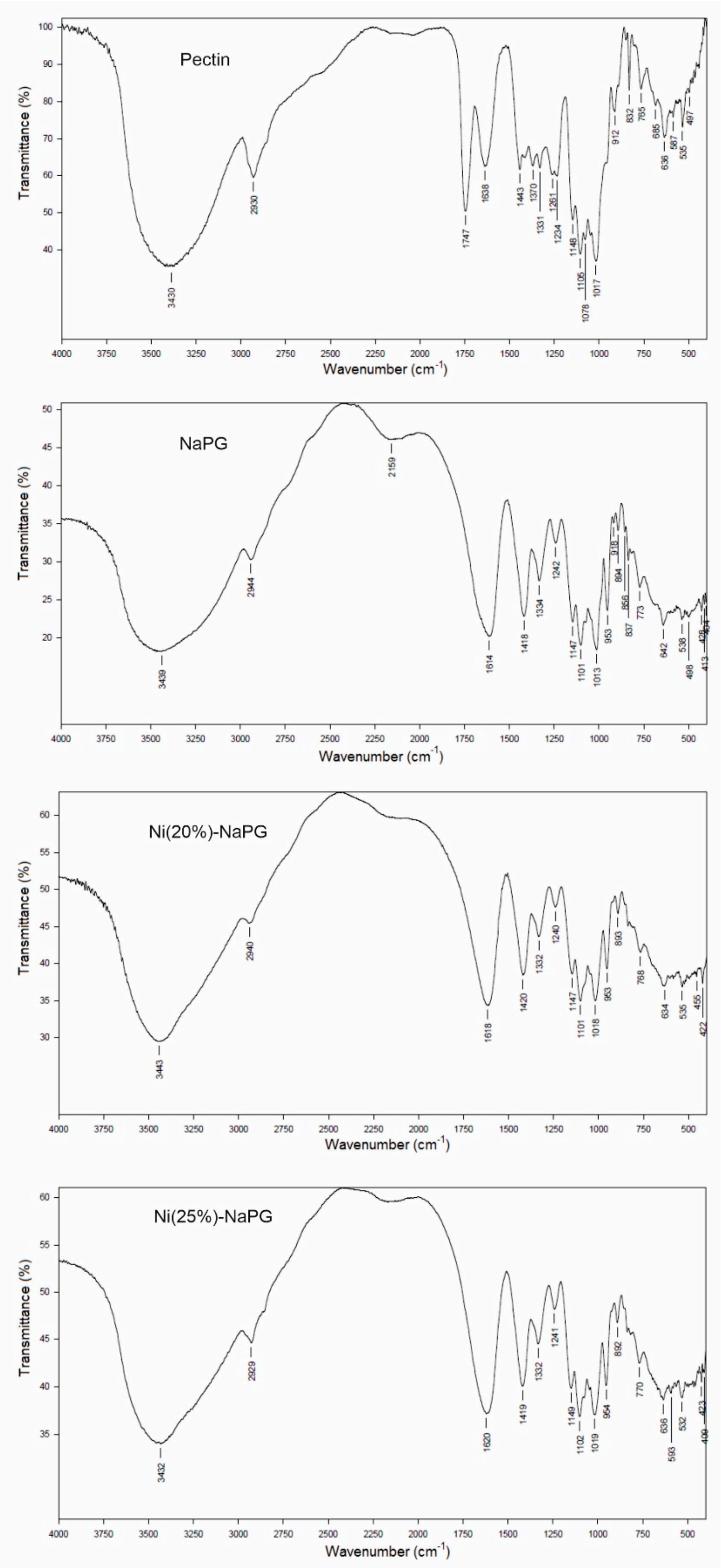

Figure S1. IR spectra of the pectin, sodium polygalacturonate (NaPG) and sodium polygalacturonate complexes [Ni(20%)-NaPG], [Ni(25%)-NaPG] with 20% and 25% substitution of sodium with nickel, consequenly.

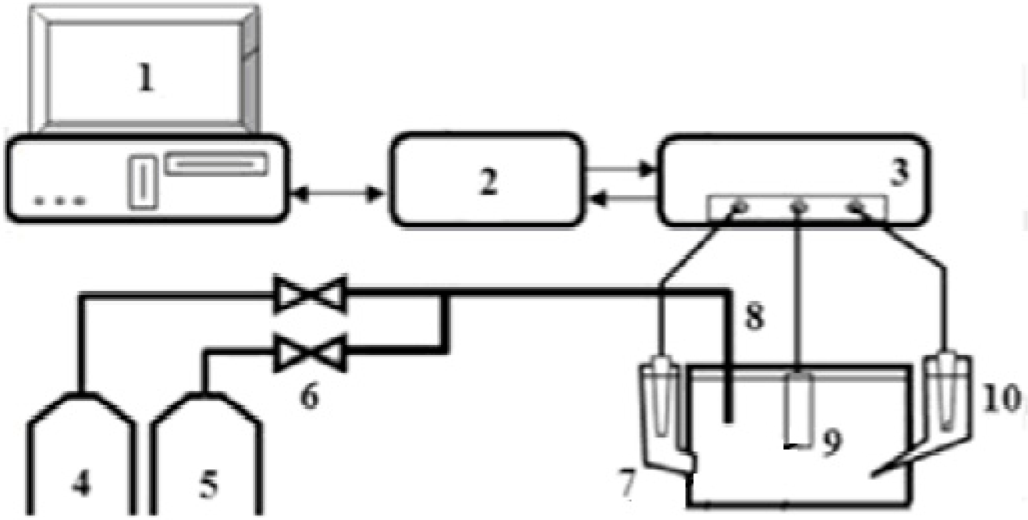

Figure S2. The scheme of electrochemical set-up. 1- computer, 2 – potentiostat Elins P-20x, 3 – rotating disc electrode – Basi RDE-2, 4 – vessel with inert gas (Ar), 5 – vessel with oxygen, 6 – valve of microadjusing, 7 – counter electrode, 8 – capillary for gas introducing, 9 – working electrode, 10 – reference electrode.

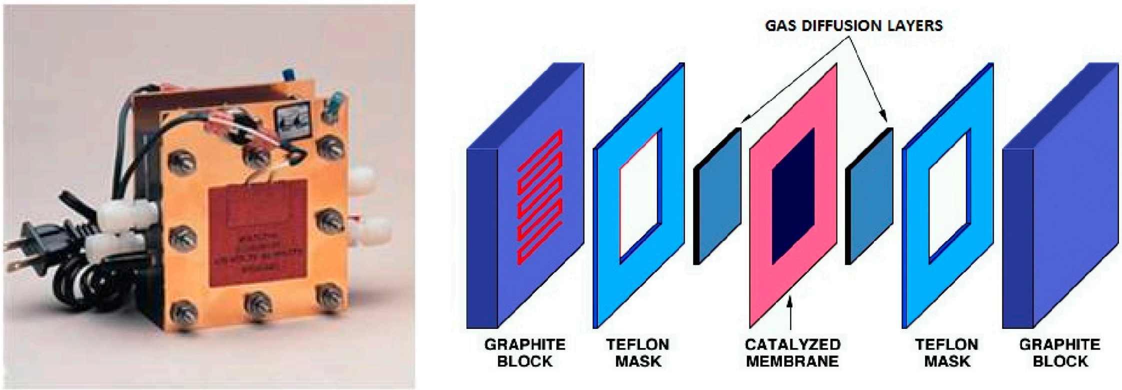

Figure S3. Scheme and appearance of the standard PEMFC used in the tests.
